# Quality of Life in Children with Β-Thalassemia Major at Center for Special Diseases 

**Published:** 2013-07-22

**Authors:** S Kaheni, M Yaghobian, G H Sharefzadah, A Vahidi, H Ghorbani, A Abderahemi

**Affiliations:** 1 MSC in Nursing, Member of faculty, Nasibeh Nursing and Midwifery faculty, Mazandaran University of Medical Sciences, Sari.; 2 Instructor, Faculty of Medicine, Birjand University of Medical Sciences, Birjand.; 3 MSc Nursing. Birjand University of Medical Sciences, Valiasr Hospital, Birjand.

**Keywords:** Quality of Life, Child, Adolescent, Blood Transfusion

## Abstract

**Background:**

Knowledge of factors associated with quality of life in patients with thalassemia is necessary for creating appropriate clinical programs, social support, and improving treatment outcomes. The purpose of this study was to determine quality of life in children with thalassemia major at Center for Special Diseases of valiasr hospital in Birjand.

**Materials and Methods:**

This cross-sectional descriptive-analytical study was conducted on 40 children over 7 years of age with thalassemia major. Tools for data collection included a demographic questionnaire and World Health Organization Quality of Life questionnaire (WHOQOL- Bref) standard questionnaire comprising 26 items to determine quality of life in patients with thalassemia. Data was analyzed using descriptive statistical tests (mean, SD, and frequency), and inferential statistical test (t-test) in SPSS software.

**Results:**

Results showed mean score of 70.37±9.88 for quality of life, 25±3.06 for physical health, 18.12±3.22 for mental health, 21.3±4.43 for living environment, and 5.95±1.58 for sociability. There was no significant correlation between quality of life and demographic variables. Correlation between social relationships and education level was significant (P-value<0.0001).

**Conclusion:**

According to the results, quality of life of the patient was above average in three dimensions of physical health, psychological health, and environmental health, and in order to improve quality of life in these children, appropriate programs should be implemented to support them physically, mentally and socially, and improve patient’s relationship with Center for Special Diseases.

## Introduction

Attention to children and adolescents is an investment for the future. Therefore, any action for attention to children and adolescents is a step toward creation of the most important structure and the best social context of the future. It is estimated that nearly 10 to 15% of children currently suffer from some kind of a chronic disease. Thalassemia major is one of these chronic diseases. Thalassemia is a chronic genetic blood disease induced by the deficiency of one or more chains of globin synthesis in hemoglobin molecule with symptoms such as: severe and chronic anemia, failure to thrive and hepatosplenomegaly, and bone disorders. Unpleasant, prolonged and repeated medication regimens in these patients can affect other aspects of their life, with severe and considerable effects on general health, psychological health and quality of life in these patients and their families (1). These patients are under various pressures like: humiliation, despair, anxiety, worries about school and employment, treatment problems, welfare issues, culture and family. There are over 20,000 people of different ages with thalassemia major in Iran. Nearly 10% of people with chronic diseases like thalassemia are adolescents. There are many reasons such as chronicity of the disease, treatment expenses, disease states, and expected early death that cause psychosocial problems in patients with thalassemia (2). These patients also have numerous difficulties in social and educational activities (3). According to Shaligram (2007) states 44% of thalassemic patient have psychiatric problems, 74% have poor quality of life, 67% of patients have symptoms of anxiety, 62% have emotional problems especially depression, and 49% have communication problems (4). According to a study by Khani et al., 47.9% of thalassemic patients had higher physical activity index of P< 0.0001, 64.9% lacked psychiatric health, 20.5% have suspected psychiatric illness, and 14.6% were healthy. Results indicated that thalassemic patients were at risk for a variety of psychiatric disorders, and consequently required experienced psychiatric consultants (5). These patients have many concerns about their disease, treatment, and health status. Older people are more prone to chronic side-effects of this disease like psychiatric fatigue, and need more psycho-social interventions (6) .Studies indicate a significant correlation between quality of life and performance level, especially educational performance in these patients (7, 8, 9, 10, 11, 12). In order to improve physical quality of life of these patients, programs aiming to provide psychological support and good communication between the patient and school authorities, family, and physician are needed. Critically sick patients with thalassemia should receive special care and attention, especially those receiving deferoxamine (11). Development of social and health policies for proper planning of prevention, diagnosis, and timely treatment of problems in different aspect of life of these patients is essential. Thus, without attention to all aspects of the disease, a better quality of life, cannot be provided for these children and their parents (5, 9). Therefore, determining quality of life of these patients leads to better understanding of their specific needs and using more effective care/treatment programs. Since quality of life had not been studied in patients with β thalassemia major in Birjand, this study was conducted to determine quality of life of children with β thalassemia major referred to Center for Special Diseases at Valiasr hospital in Birjand in 2010.

## Materials and Methods

This cross-sectional descriptive analytical study was conducted on 40 children over 7 years of age with thalassemia major. Children under study depended on blood transfusion referred to Center for Special Diseases at Valiasr Hospital in Birjand to receive their monthly blood transfusion. During 21 April 2011 to 21 June 2012 of all children with thalassemia major who were admitted to this center, children over 7 years of age which have to receive blood transfusion on monthly basis at Valiasr Hospital in Birjand, were selected for the study by census. For data collection, questionnaires were used. The researcher-made demographic questionnaire included age, gender, occupation, education, severity of medication complication, craniofacial changes, type of medication used, frequency of medication per week, and splenectomy. Furthermore, the World Health Organization standard quality of life questionnaire WHOQOL (Bref) was used. The short form WHOQOL-Bref questionnaire assesses quality of life in general. This questionnaire has been translated and validated in over 40 countries (13, 14, and 15). WHOQOL-Bref assesses four domains of physical health, psychological health, social relationships, and environment with 24 items. Each of these domains has 7, 6, 3, and 8 items, respectively. The first two items were not related to any of these domains, and assessed health and quality of life in general, adding up to 26 items in total. After performing necessary calculations for of each domains separately, scores 4to20 were achieved that 4 represents worst and 20 the best domain. These points are convertible to a score range 0-100. Reliability of the questionnaire for the present study was found through Cronbach’s alpha 0.9. Each item assessed subject’s status in the past two weeks on a 5-option Likert scale (never= 1, low= 2, moderate= 3, high= 4, very high= 5). It should be noted that the physical health domain was concerned with issues like mobility, daily routine activities, work capacity and energy, pain, and sleep. Psychological health domain addressed appearance, negative feelings, positive feelings, self-confidence, thoughts, learning, memory and concentration, and religion and spirituality. In the social relationship domain, personal relationships, social support, and sex life were questioned. Questions about environmental health were concerned with financial resources, physical security, physical environment of the habitat, opportunities for acquiring skills, new information, recreation, physical environment (noise and air pollution), and transportation (15). Questionnaires were written through interviews and completed by trained students. Explanations about the disease and reasons for conducting this study were given to the patients, and permission to complete the questionnaire was obtained from the patient and parents. Literate patients were asked to complete the questionnaire themselves if they wished. Considering the young age of the patients, questions were asked, and interviews were conducted in such a way so as not to undermine integrity and independence of questions.

**Statistical Analysis**

Data was analyzed using descriptive statistics (frequency, mean, and standard deviation) and inferential statistics (t-test) in SPSS-16 software.

## Discussion

In the present study, mean score of quality of life was found 70.37. Also, mean physical health score was 25, mean psychological health score was 18.12, mean environment health was 21.3, and mean social relationship score was 5.95. Considering the results obtained, quality of life of the subjects was above average in three dimensions of physical health, psychological health, and environmental health. However, in social relationship dimension, quality of life was less than average. One of the reasons for quality of life being less than medium in this dimension can be the low number of items in this dimension. In a study by Yousefi et al (11), on quality of life in patients with thalassemia and their families, (according to WHOQOL-Bref questionnaire) mean physical health score was 3.43, mean psychological health score was 3.4, mean environmental health score was 3.55, and mean social relationship score was 2.6 (in this study, means were divided by number of items in each dimension), which agrees with the results obtained in this study. In another study by Khani et al(5), quality of life of patients with thalassemia major in southern coast of the Caspian sea, (according to the short form of SF-36 questionnaire) reported favorable quality of life in the domain of physical health that is in accordance with results found in this study, but they reported lack of health in psychological health domain. The higher scores in dimensions of quality of life in the present study is indicative of more support from the Center for Special Diseases in this region, as well as better and more effective cooperation of families in care of these patients in different dimensions. In this study, age was investigated as an influential factor in quality of life, but showed no significant correlation. In a study by Tahmasebi et al (9) no significant correlation was found between patients’ age and quality of life. However, in a study by Thavorncharoensap et al(16) the correlation between age and quality of life was found significant (P-value<0.05). In the present study, no significant correlation was observed between quality of life and occupation in patients. In Khani et al (5) study, this correlation was significant (P-value< 0.05). Also, in the present study, education level and its correlation with quality of life was investigated and education level (0.03) and social relationships were found significantly correlated (P= 0.0001). In a study by Dahlui et al(17), a significant correlation was observed between education level and quality of life (P< 0.05). The next variable considered in this study was clinical complications and their correlation with quality of life, which was found insignificant. In Shaligram et al (12) study, correlation between quality of life and clinical complications was reported significant (P-value< 0.05). Also, correlation between severity of clinical complications and quality of life was reported significant by Thabvorncharoensap et al(16). In the present study, craniofacial changes as an effective factor in quality of life was investigated, and their correlation was found insignificant, which could be due to the low sample size. Another variable of the study was type of medication, which was not significantly correlated with quality of life. In the study by Thavorncharoensap et al (16),a negative significant correlation was found with iron chelation therapy (P-value< 0.05). In the present study, correlation between quality of life and frequency of medication per week was investigated, and an insignificant correlation was found between the two groups. Tahmasebi et al (9) showed frequency of medication had led to reduce general quality of life in children, especially in social relationship dimension. In the present study, no significant correlation was found between splenectomy and quality of life, as in the study by Tahmasebi et al. In Dahlui et al (17) study, correlation between splenectomy and quality of life was insignificant (P-value= 0.058). Study limitations included occurrence of unforeseeable events that affected patients’ responses during interviews and completion of questionnaires. Also, patients’ refusal to openly express their opinions, unwillingness to cooperate due to considering this type of study useless for improving quality of life of these patients, travel fatigue, weakness and lack of energy and subsequent lack of patience to answer questions could be cited as other limitations of the study.

## Conclusion

Results indicated that the quality of life of patient in three dimensions of physical, psychological, and environmental health was above average, but in the dimension of social relationships, quality of life was below average. Craniofacial changes as an effective factor in quality of life was investigated. It is recommended that relevant organizations, by paying more attention to different aspects of life of these patients help to improve their quality of life in all dimensions. Furthermore, it is necessary to improve patients’ and their families’ awareness, and to use experts.
